# The relationship of hair glucocorticoid levels to immunological and virological outcomes in a large cohort of combination antiretroviral therapy treated people living with HIV

**DOI:** 10.1186/s12879-022-07257-x

**Published:** 2022-03-20

**Authors:** Quan Zhang, Xiaoming Li, Shan Qiao, Shuaifeng Liu, Yuejiao Zhou, Zhiyong Shen

**Affiliations:** 1 South Carolina SmartState Center for Healthcare Quality (CHQ), University of South Carolina Arnold School of Public Health, Columbia, SC 29208 USA; 2 Integrative Muscle Biology Laboratory, Division of Rehabilitation Sciences, College of Health Professions, University of Tennessee Health Science Center, Cancer Research Building, Room 335, 19S Manassas Street, Memphis, TN 38103 USA; 3 Guangxi Zhuang Autonomous Region Center for Disease Control and Prevention, No.18 Jinzhou Road, Nanning, Guangxi 530028 China

**Keywords:** Hair cortisol, Hair cortisone, CD4 count, HIV viral load, People living with HIV

## Abstract

**Background:**

Existing literature mostly investigated the relationship of acute or short-term glucocorticoid exposure to HIV disease progression using cortisol levels in serum, saliva, or urine. Data are limited on the relationship of long-term glucocorticoid exposure to HIV disease progression. This study examined whether hair glucocorticoid levels, novel retrospective indicators of long-term glucocorticoid exposure, are associated with two common indicators of HIV disease progression (CD4 count and HIV viral load) among a large cohort of combination antiretroviral therapy treated Chinese people living with HIV (PLHIV).

**Methods:**

A total of 1198 treated PLHIV provided hair samples for glucocorticoid (cortisol and cortisone) assay and completed a survey assessing sociodemographic, lifestyle, and HIV-related characteristics. Meanwhile, CD4 count and HIV viral load were retrieved from their medical records. Spearman correlation was used to examine the associations of hair cortisol and cortisone levels to continuous CD4 count and HIV viral load. Multivariate logistic regression was used to predict CD4 count < 500 cells/mm^3^.

**Results:**

Both hair cortisol and cortisone levels were negatively associated with CD4 count but not with HIV viral load. The odds ratio for CD4 count < 500 cells/mm^3^ was 1.41 [95% CI 0.99–2.00] and 2.15 [95% CI 1.51–3.05] for those with hair cortisol and cortisone levels in the highest quartile compared to the lowest when controlling for sociodemographic, lifestyle, HIV-related covariates, and HIV viral load.

**Conclusion:**

Hair glucocorticoid levels were associated with CD4 count but not viral load in treated Chinese PLHIV. Our data furtherly supported the hypothesis that elevated glucocorticoid levels are associated with the lower CD4 count.

## Background

With the help of combination antiretroviral therapy (cART), HIV has shifted from a terminal illness with high mortality to chronic disease [1], and people living with HIV (PLHIV) have a nearly normal life expectancy when given access to adequate cART [2]. However, HIV disease progression on an individual basis varies widely. While the HIV disease progression is influenced by various factors, activation of the hypothalamic–pituitary–adrenal (HPA) axis, resulting in elevated glucocorticoid levels, has been proposed as one of the critical pathways to hasten HIV disease progression. Theoretically, high glucocorticoid levels could induce T-lymphocyte production of cytokines, trigger the destruction of CD4 T cell (CD4) through programmed cell death and bolster HIV viral replication [3, 4].

Several studies have examined whether cortisol (active glucocorticoid) levels are associated with indicators of HIV disease progression among PLHIV. However, the findings are inconsistent, with some studies found that elevated cortisol levels were associated with indicators of HIV disease progression [5–11], whereas other studies suggested no such association [12–20]. Previous studies used acute or short-term cortisol levels in liquid biological samples (e.g., serum, saliva, or urine). These measurements reflect cortisol levels of that specific time window varying from a few minutes (single-point blood and saliva sample) up to a maximum of 24 h (multi-point saliva and urine sample), which may not well-match the etiology of chronic disease progression. In addition, cortisol levels in these liquid biological samples showed significant circadian rhythm and were easily affected by state reactivity. Recently, several studies used cortisol levels in hair among PLHIV [21–24]. In comparison with cortisol levels in liquid biological samples, cortisol levels in hair can better estimate long-term cortisol exposure (weeks to months) [25]. To the best of our knowledge, only two published studies reported additional data about the relationship between hair cortisol levels and two common indicators of HIV disease progression (CD4 count and HIV viral load), and a nonsignificant association was found [21, 23]. The two previous studies are limited in enrolling small sample size participants and not considering some potential factors related to hair cortisol levels (e.g., hair washing). Thus, a study of hair cortisol levels and HIV disease progression in a large cohort of PLHIV is warranted, further considering potential confounders.

Recently, hair cortisone levels have been served as a useful additional biomarker for assessing long-term glucocorticoid exposure. As well known, circulating cortisol levels are dynamically regulated partially through the activity of the 11β hydroxysteroid dehydrogenase (11β-HSD) enzyme, where cortisol is converted to cortisone by the activity of 11β-HSD type 2 enzyme, and cortisone can be regenerated from cortisol by the activity of 11β-HSD type1 enzyme [26]. Therefore, cortisol and cortisone could regulate physiological and psychological responses together [27, 28]. However, it is unknown about hair cortisone levels in PLHIV and their relationship with indicators of HIV disease progression.

Accordingly, to extend knowledge on the associations of long-term glucocorticoid levels with HIV disease progression, we assessed hair cortisol and cortisone levels and two common indicators of HIV disease progression (CD4 count and HIV viral load) in a large cohort of cART treated Chinese PLHIV in Guangxi, China. We aimed to examine whether hair cortisol and cortisone levels were associated with those indicators of HIV disease progression when controlling for potential confounders, including sociodemographic, lifestyle, and HIV-related characteristics.

## Methods

### Participants

Participants were recruited from November 2017 to February 2018 as part of a longitudinal HIV stigma project, which aimed to investigate the effect of HIV-related stigma on clinical outcomes in Guangxi, China [29, 30]. In collaboration with the Guangxi center for disease control and prevention (CDC), PLHIV, who aged 18 years, willing to provide hair samples and retrieve virologic and immunological outcomes from their medical record, were eligible for participation. PLHIV with any of the following characteristics were excluded: (1) linguistic, mental or physical inability to respond to assessment questions; (2) opportunistic infections or endocrine diseases (e.g., Cushing’s syndrome); (3) currently taking hormonal medications (e.g., oral contraceptive) or known history of illegal drug use; (4) chemical hair treated (e.g., dyed, permed, or bleached) or scalp hair in the posterior vertex was less than 1 cm.

Medical staff or HIV case managers at the study sites referred potential participants to the research team members. PLHIV were checked for eligibility, informed of the study's benefits and risks, and invited to the study when they finished their routine visit. Research team members were local CDC staff or health care workers in the HIV clinics who had received intensive training on research ethics and interview skills with PLHIV before the field data and hair specimen collection. Finally, a total of 1198 PLHIV participated in this study.

### Hair and data collection

Hair samples were cut from the posterior vertex region as close as possible to the scalp following a standard protocol [31, 32] in private rooms of local CDC or HIV clinics. The hair thatch then was completely enclosed by a piece of foil, and a small label indicating the study ID number was placed over the distal end of the hair thatch. Then, the interviewer-administered questionnaire was used for the collection of sociodemographic and lifestyle information. Each participant received compensation for their time spent to respond to the questionnaire and to provide hair samples with a value equal to $5.00 (≈ 35 Chinese Yuan) after finishing the survey.

### Hair cortisol and cortisone

The proximal 1 cm of hair segments (approximately reflecting the last month of accumulative cortisol and cortisone levels) was cut finely with scissors, and 20 mg processed and analyzed using liquid chromatography-tandem mass spectrometry (LC/MS/MS) followed the protocol described by Gao and colleagues [33]. The method showed excellent linearity (R^2^ > 0.99) in the range of 0.5–1250 pg/mg for both cortisol and cortisone, the limits of detection and quantitation at 0.2 pg/mg and at 0.5 pg/mg for both cortisol and cortisone, respectively. Intra-day and inter-day percentage coefficients of variation were less than 5% at standard concentrations of 1.25, 25, and 250 pg/mg, and recovery ranged between 95 and 107% for both cortisol and cortisone.

### CD4 count and HIV viral load

CD4 count and HIV viral load data were extracted from their clinical records after their routine visit. Given that CD4 count and HIV viral load data are generally skewed, CD4 count was dichotomized as ≥ 500 cells/mm^3^ vs. < 500 cells/mm^3^ because the low limit of CD4 count is 500 cells/mm^3^ in healthy adults. HIV viral load was dichotomized as ≥ 200 copies/ml vs. < 200 copies/ml because the “Undetectable = Untransmittable” initiative defined undetectable viral load as viral load less than 200 copies/ml that cannot sexually transmit HIV to others [34].

### Sociodemographic, lifestyle, and other HIV-related characteristics

Participants provided information on their sociodemographic and lifestyle characteristics that might have a potential influence on hair cortisol and cortisone levels [35–37], including age, gender, ethnicity, marital status, education level, employment status, monthly household income level, height, weight, smoking status, drinking status and frequency of hair washing. Body Mass Index (BMI) was calculated using an established formula BMI = weight (kg)/ [height (m)]^2^ based on weight and height for each participant.

Years of HIV diagnosis, current cART status (first-line vs. second-line), and years of current cART were also extracted from their clinical records. cART adherence (≥ 95% vs. < 95%) was measured using the single-item question: “In last month, how many days were you able to take your medications exactly as prescribed?” Adherence was calculated by dividing the reported number of days by 30 days [38].

### Statistical analyses

Of the 1198 PLHIV, one participant was excluded due to insufficient weight of hair samples (less than 20 mg) for cortisol assaying, and six participants were excluded because of unavailable hair cortisol levels due to the analysis mistake, leaving an effective sample of 1191 participants for analysis.

As indicated by the Kolmogorov–Smirnov test, continuous hair cortisol, hair cortisone, CD4 count, or HIV viral load was not the normal distribution, then Spearman’s correlation was used to examine the associations of continuous hair cortisol and hair cortisone levels with continuous CD4 count and HIV viral load. Logistic regression models were used to estimate the association of hair cortisol and cortisone levels with CD4 count < 500 cells/mm^3^ but not HIV viral load ≥ 200 copies/ml because the number of viremic participants is very low (n = 35, 2.9%). Hair cortisol and cortisone levels quartiles were calculated, and the second, third, and fourth quartiles were contrasted with the first quartile as the reference category. Crude odds ratios (cOR) and their 95% confidence interval (95%CI) were reported for univariate analysis. Adjusted odds ratios (aOR) and their 95% CI were derived from multivariate analyses after controlling for sociodemographic, lifestyle, and HIV-related characteristics, including HIV viral load. All analyses were performed using SPSS for Windows, version 26 (IBM, Chicago, Illinois).

## Results

Of the 1191 participants with a median age of 38 years (Table 1), 64.3% were male, 65.1% were Han ethnicity, 56.6% were married, 81.6% were employed, 67% were never smoking, 50.3% were never drinking, 88.2% were washing their hair less than four times per week, 79.7% were receiving the first-line cART, and 92.8% reported optimal cART adherence (≥ 95%), 2.9% had HIV viral load ≥ 200 copies/ml. Most of the participants had low income and education levels, with 60.3% reporting a monthly household income of less than 3000 Chinese Yuan (or approximately $460 during the survey) and 60.2% reporting not finishing junior high school. The median BMI was 21.29 kg/m^2^. The median years of HIV diagnosis and years of cART were 5.33 and 4.42 years, respectively.

**Table 1:** Distribution of variables in the participants (n = 1191)

Variables	n (%)/ median (range)
Age, years	38 (18–60)
18–30	197 (16.5%)
31–40	493 (41.4%)
41–50	323 (27.1%)
51–60	178 (14.9)
Gender	
Male	766 (64.3%)
Female	425 (35.7%)
Ethnicity	
Han	775 (65.1%)
No-Han	416 (34.9%)
Marital status	
Married	674 (56.6%)
Others	517 (43.4%)
Education levels	
≤ 9 years	717 (60.2%)
> 9 years	474 (39.8%)
Employment status	
Employed	972 (81.6%)
Unemployed	219 (18.4%)
Monthly income level	
≥ 3000 Yuan	473 (39.7%)
< 3000 Yuan	718 (60.3%)
BMI, kg/m^2^	21.29 (13.11–31.55)
Smoking	
Yes	393 (33.0%)
No	798 (67.0%)
Drinking	
Yes	592 (49.7%)
No	599 (50.3%)
Frequency of hair washing, times/week	
≥ 4 times/week	141 (11.8%)
< 4 times/week	1050 (88.2%)
Years of HIV diagnosis	5.33 (0.25–14.67)
cART status	
First-line cART	949 (79.7%)
Second-line cART	242 (20.3%)
Years of cART	4.42 (0.25–13.00)
Self-reported adherence in the past month	
≥ 95%	1105 (92.8%)
< 95%	86 (7.2%)
Hair cortisol, pg/mg	28.17 (0.20–3587.90)
Hair cortisone, pg/mg	27.45 (0.20–1893.17)
CD4 count, cells/mm^3^	470 (8–1685)
≥ 500 cells/mm^3^	536 (45.0%)
< 500 cells/mm^3^	655 (55.0%)
HIV viral load, copies/ml	
≥ 200 copies/ml	35 (2.9%)
< 200 copies/ml	1156 (97.1%)

*BMI* body mass index, *cART* combination antiretroviral therapy

The median hair cortisol levels were 28.17 pg/mg and hair cortisone levels were 27.45 pg/mg. As presented in Table 2, spearman’s correlation revealed that hair cortisol levels and hair cortisone levels were positively correlated (*r* = 0.226, *p* < 0.001); hair cortisol and cortisone levels were negatively correlated with CD4 count (*r* = − 0.129, *p* < 0.001 for hair cortisol levels; *r* = − 0.148, *p* < 0.001 for hair cortisone levels); neither hair cortisol nor cortisone levels were correlated with HIV viral load (*r* = 0.025, *p* = 0.395 for hair cortisol levels; *r* = − 0.025, *p* = 0.396 for hair cortisone levels).

**Table 2:** Correlation between hair cortisol, hair cortisone, CD4 count, and HIV viral load

	Hair cortisol	Hair cortisone	CD4 count	HIV viral load
Hair cortisol	–			
Hair cortisone	0.226***	–		
CD4 count	− 0.129***	− 0.148***	–	
HIV Viral load	0.025	− 0.025	0.070*	–

* < 0.05; *** < 0.001

Of 1191 PLHIV, 55.0% had CD4 count < 500 cells/mm^3^ (see Table1). The results of logistic regression models of CD4 count < 500 cells/mm^3^ are presented in Table 3. The cORs were 1.77 [95% CI 1.28–2.45] and 2.39 [95% CI 1.72–3.33] for those with hair cortisol and cortisone levels in the highest quartile compared to the lowest quartile in univariate analysis. The aORs were 1.41 [95% CI 0.99–2.00] and 2.15 [95% CI 1.51–3.05] for those with hair cortisol and cortisone levels in the highest quartile compared to the lowest quartile when controlling for sociodemographic, lifestyle, and HIV-related characteristics.

**Table 3:** Logistic regression models of CD4 count < 500 cells/mm^3^

	n (%)	Univariate		Multivariate	*p*
		cOR (95% CI)	*p*	aOR(95% CI)	
Hair cortisol					
First quartile	146 (49.0%)	1 [reference]	–	1 [reference]	–
Second quartile	157 (52.7%)	1.18 (0.85–1.62)	0.326	1.14 (0.82–1.60)	0.444
Third quartile	166 (55.7%)	1.33 (0.96–1.83)	0.085	1.10 (0.78–1.55)	0.596
Fourth quartile	186 (62.6%)	1.77 (1.28–2.45)	< 0.001	1.41 (0.99–2.00)	0.055
Age					
18–30	–	–	–	1 [reference]	–
31–40	–	–	–	1.08 (0.74–1.58)	0.684
41–50	–	–	–	1.87(1.21–2.88)	0.005
51–60	–	–	–	2.05(1.26–3.32)	0.004
Gender	–	–	–	0.65 (0.47–0.90)	0.009
Ethnicity	–	–	–	0.72 (0.56–0.93)	0.01
Marital status	–	–	–	0.99 (0.76–1.30)	0.961
Education levels	–	–	–	0.59 (0.45–0.78)	< .001
Employment status	–	–	–	0.91 (0.66–1.25)	0.547
Monthly income levels	–	–	–	0.98 (0.76–1.27)	0.874
BMI	–	–	–	0.95 (0.91–0.99)	0.017
Smoking	–	–	–	0.87 (0.65–1.18)	0.369
Drinking	–	–	–	0.83 (0.63–1.09)	0.172
Frequency of hair washing	–	–	–	0.84 (0.57–1.24)	0.381
Years of HIV diagnosis	–	–	–	0.90(0.81–0.97)	0.01
cART status	–	–	–	1.08 (0.79–1.47)	0.627
Years of cART	–	–	–	1.04 (0.95–1.14)	0.408
Self-reported adherence	–	–	–	1.26 (0.79–2.01)	0.323
HIV viral load	–	–	–	2.03 (0.93–4.42)	0.076
Hair cortisone					
First quartile	132 (44.4%)	1 [reference]	–	1 [reference]	–
Second quartile	162 (54.2%)	1.48 (1.07–2.04)	0.018	1.52 (1.09–2.13)	0.014
Third quartile	165 (55.4%)	1.55 (1.12–2.14)	0.008	1.50 (1.07–2.12)	0.020
Fourth quartile	195 (65.7%)	2.39 (1.72–3.33)	< 0.001	2.15 (1.51–3.05)	< 0.001
Age					
18–30	–	–	–	1 [reference]	–
31–40	–	–	–	1.10 (0.75–1.61)	0.631
41–50	–	–	–	1.83(1.18–2.82)	0.007
51–60	–	–	–	1.98(1.21–3.23)	0.006
Gender	–	–	–	0.62 (0.45–0.85)	0.003
Ethnicity	–	–	–	0.73 (0.57–0.94)	0.016
Marital status	–	–	–	1.00 (0.77–1.31)	0.992
Education levels	–	–	–	0.61 (0.46–0.80)	< .001
Employment status	–	–	–	0.89 (0.64–1.23)	0.469
Monthly income levels	–	–	–	1.01 (0.78–1.31)	0.934
BMI	–	–	–	0.95 (0.91–0.99)	0.009
Smoking	–	–	–	0.85 (0.63–1.15)	0.287
Drinking	–	–	–	0.85 (0.65–1.12)	0.244
Frequency of hair washing	–	–	–	0.84 (0.57–1.24)	0.37
Years of HIV diagnosis	–	–	–	0.88 (0.81–0.96)	0.006
cART status	–	–	–	1.10 (0.80–0.96)	0.554
Years of cART	–	–	–	1.04 (0.95–1.14)	0.403
Self-reported adherence	–	–	–	1.25 (0.78–2.00)	0.349
HIV viral load	–	–	–	2.11 (0.96–4.63)	0.063

*CI* confidence interval, *cOR* crude odds ratio, *aOR* adjusted odds ratio, Adjusted for age, gender, ethnicity, marital status, education level, employment status, monthly income levels, BMI, smoking, drinking, frequency of hair washing, years of HIV diagnosis, cART status, years of cART, self-reported adherence, and HIV viral load in the multivariate model

## Discussion

The central question investigated in this study was whether hair cortisol and cortisone levels, novel retrospective indicators of long-term glucocorticoid exposure, were associated with two common indicators of HIV disease progression (CD4 count and HIV viral load) among a large cohort of Chinese PLHIV. We found that hair glucocorticoid levels were negatively associated with CD4 count but not HIV viral load.

Notably, we found that hair glucocorticoid levels were negatively associated with CD4 count and the aORs for CD4 count < 500 cells/mm^3^ were 1.41 times and 2.15 times for those with hair cortisol and cortisone levels in the highest quartile compared to the lowest quartile. The result regarding the significant association between hair cortisol levels and CD4 count was in line with part of previous studies that also reported a negative association between CD4 count and cortisol levels in plasma and saliva [5–9] but differed from the results from some published studies in which a nonsignificant association was reported by using serum cortisol levels [15, 18–20], urinary cortisol levels [12, 13, 16, 17], or hair cortisol levels [21]. Interestingly, we found stronger associations between hair cortisone levels and CD4 count than hair cortisol levels did, which was in line with some previous studies that also reported stronger associations between hair cortisone levels and variables studied than hair cortisol levels did, including Parkinson’s disease [39], Cushing’s syndrome [40], cardiometabolic variables [41, 42], and stress-related variables [39, 43]. In addition, we found hair cortisone levels and hair cortisol levels were positively correlated, which is similar to the findings in previous studies [39, 41, 42, 44–46]. Previous studies indicated that salivary cortisone levels were more closely associated with unbound, biologically active cortisol levels than total cortisol levels [26], and hair cortisone levels were significantly associated with salivary cortisone levels [45, 47]. Therefore, hair cortisone levels may provide a valuable surrogate for assessing long-term free cortisol levels. Our findings with large sample size and better controlling potential confounders could further support the hypothesis that elevated glucocorticoid levels are associated with CD4 declining and provide implications for future research to consider both hair cortisol and cortisone levels to reflect the long-term glucocorticoid exposure. Furthermore, previous studies indicated linkages among gut dysbiosis, leaky gut, CD4 count, and cortisone in the HIV-related research field [48–51]. Future research could explore those linkages using hair levels of cortisol and cortisone.

We found that neither hair cortisol levels nor hair cortisone levels were associated with HIV viral load. Our results regarding the nonsignificant association between hair glucocorticoid levels and HIV viral load were generally in line with previous cross-sectional studies by employing hair cortisol levels [21, 23], serum cortisol levels [14, 52], and urinary cortisol levels [16]. However, one longitudinal study reported that urinary cortisol levels could predict a faster increase in HIV viral load over 4 years [12]. Thus, further research is needed to explore this association with a longitudinal design.

While our study does hold some strengths, including enrolling a large cohort of PLHIV as participants, controlling various confounders, and using 1-cm hair to measure the past month's glucocorticoid exposure, several limitations need to be acknowledged. First, the current study was based on cross-sectional data. Our findings cannot draw causal inferences. Future research should examine the predictive value of hair glucocorticoid levels for incident HIV disease progression using longitudinal designs. Second, several other glucocorticoid measurements (e.g., cortisol awakening response and diurnal slope) in blood or saliva [8, 53], cortisol regulations (e.g., the sensitivity of the glucocorticoid receptor) [54–56], and other indicators of HIV disease progression (e.g., HIV DNA levels, CD8 and CD16) should be considered in future research in that they may yield robust conclusions. Third, because all participants are from Guangxi, China, these findings may not be generalizable to other PLHIV settings. Fourth, while data were not available in the current study on some other potential factors (e.g., physical exercise, depression, and some other medications) that might influence hair cortisol levels, CD4 count, and HIV viral load [35, 57], those factors should be considered in future research.

## Conclusion

In summary, this study found that hair cortisol and cortisone levels were negatively associated with CD4 count, but not HIV viral load, in a large cohort of Chinese PLHIV. Our data furtherly supported the hypothesis that elevated cortisol levels are associated with lower CD4 counts. Future work will focus on the longitudinal relationship of cortisol and cortisone levels in various biological samples to indicators of HIV disease progression.

## Data Availability

The datasets generated and/or analyzed in the current study are available from the corresponding author on reasonable request.

## Abbreviations

11β-HSD: 11β Hydroxysteroid dehydrogenase
BMI: Body mass index
cART: Combination antiretroviral therapy
CD4: CD4 + T-lymphocytes
CDC: Center for disease control and prevention
CI: Confidence interval
HIV: Human immunodeficiency virus
HPA: Hypothalamic–pituitary–adrenal
LC/MS/MS: Liquid chromatography-tandem mass spectrometry
OR: Odds ratio
aOR: Adjusted odds ratio
cOR: Crude odds ratio
PLHIV: People living with HIV
SPSS: Statistical product and service solutions
